# Does Pre-Operative Grade of Stress Urinary Incontinence Severity Affect the Post-Operative Outcome? A Systematic Review

**DOI:** 10.1007/s00192-025-06275-y

**Published:** 2025-09-24

**Authors:** Themistoklis Mikos, Nikolaos Roussos, Iakovos Theodoulidis, Christos Anthoulakis, Grigoris F. Grimbizis

**Affiliations:** 1st Department of Obstetrics and Gynecology, Aristotle University of Thessaloniki, Papageorgiou General Hospital, Periferiaki Odos Thessalonikis, 56403 Thessaloniki, Nea Efkarpia Greece

**Keywords:** Evaluation tools, Severity, Stress urinary incontinence, Surgery, Tension-free vaginal tape

## Abstract

**Introduction and Hypothesis:**

It is not known whether assessment of severity of stress urinary incontinence (SUI) is important before any attempt at the correction of it. The aim of this study is to identify the success rates and/or dry rates following interventional treatment in patients with mild, moderate, and severe SUI, and compare the results among the various treatments.

**Methods:**

This systematic review was conducted according to Preferred Reporting Items for Systematic Reviews and Meta-Analyses guidelines and registered in the Prospective Register of Systematic Reviews System (ID: CRD420251017900), by searching PubMed, Scopus, and Cochrane Library Database from inception to December 2024. The Population, Intervention, Comparison, Outcomes, and Study design criteria were as follows: the ‘Population’ were adult women who underwent an interventional procedure for SUI and had pre- and post-interventional classification of SUI severity. As ‘Intervention’ was considered any type of interventional procedure: colposuspensions, mid-urethral slings (MUS), pubo-vaginal slings, energy-based devices (EBD), and injectables. ‘Comparison’ was considered between either single or comparative interventions according to the pre- and post-operative grade of SUI severity. ‘Outcomes’ were the success rates and/or dry rates according to pre- and post-operative grade of SUI severity.

**Results:**

From a total of 11,535 studies, 24 (4380 patients) were included for further analysis: in 13 studies (*n* = 3599) a graft was used and in 11 studies (*n* = 781) other interventions. Successful treatment of mild incontinence was achieved in 84.7% (MUS = 89.6%, EBD = 66.7%, *p* < 0.001), of moderate incontinence in 88.3% (MUS = 92.0%, EBD = 52.5%, *p* < 0.001), and of severe/very severe incontinence in 75.7% (MUS = 83.9%, EBD = 44.3%, *p* < 0.001).

**Conclusions:**

The success rates of any incontinence procedure depend largely on the pre-operative severity of SUI, and they are significantly lower with increasing severity of the SUI. MUS appear to have improved treatment rates compared with EBD, independently of the severity of SUI.

**Supplementary Information:**

The online version contains supplementary material available at 10.1007/s00192-025-06275-y

## Introduction

### Rationale

The severity of stress urinary incontinence (SUI) can be evaluated using a variety of instruments [1]. For many decades and before the introduction of the current recommendations about no use of urodynamics prior to uncomplicated SUI surgery, SUI was mainly classified as type I (descent of the bladder base), type II (descent of the bladder base and rotation of the urethra), and type III (no descent of the bladder base) [2]. The severity of SUI was further classified according to abdominal/Valsalva leak point pressure (ALPP/VLPP) to grade 1 (ALPP > 90 cmH_2_O), grade 2 (ALPP 60–90 cmH_2_O), and grade 3 (ALPP < 60 cmH_2_O) [3]. Based on these assumptions, Ford and Ogah systematically reviewed the literature and concluded that the patients with grade 1 SUI enjoy higher rates of subjective cure after mid-urethral sling (MUS) procedures [4]. Therefore, it appears that it is already known that severe SUI demands a more aggressive surgical approach to obtain satisfactory results.

However, urodynamics are no longer a pre-operative necessity, so the McGuire classification of SUI is not a prerequisite for the uncomplicated SUI patient, as the office evaluation alone is shown to be not inferior to evaluation with urodynamic testing for clinical outcomes at 1 year [5, 6]. Thus, although severity of SUI is a parameter that is well accepted in the nomenclature of the investigation and treatment of this condition, current practice does not necessitate the pre-operative evaluation of the severity of SUI [5]. Similarly, the classification of the patients by severity both prior to and after an intervention for SUI is almost universally absent from the relevant literature [1].

In most of the high-quality, contemporary studies, pre- and post-operatively nonvalidated cough stress test, urodynamics, and the 1-h pad test are used for the objective evaluation of the severity of SUI, whereas standardized questionnaires such as the Incontinence Impact Questionnaire (IIQ-7), the Urinary Distress Inventory (UDI-6), and the International Consultation on Incontinence Questionnaire (ICIQ), are used for the subjective evaluation of the severity of SUI [1]. This is not in line with older literature, where Stamey’s scoring system (grade 0 = no incontinence, grade 1 = incontinence at coughing or straining, grade 2 = incontinence at changing position or walking, and grade 3 = incontinence at all times) or Sandvik Severity Index were mostly used [7–9]. More importantly, there are very few contemporary studies that display their results according to the pre-operative severity of SUI. Therefore, the available information regarding the results of different treatments on different grades of SUI remains obscure and subjective [1]. Thus, currently it is difficult to answer whether it is important to define the pre-operative severity of SUI based on the routine investigations that are suggested for the evaluation of the patients.

Moreover, it is not known whether the assessment of the severity of SUI is important before any attempt for the correction of it. It is widely accepted that if a treatment offers certain success in cure rates, these rates apply to all the patients with SUI, irrespective of the severity of the condition. This may be true; however, different treatments can be associated with different kinds of complications, and it could be more logical to decide which treatment to apply to each patient with a strategy of maximum results with minimum complications. Consequently, common sense suggests applying the one with the less significant complications among equally effective treatments.

In the current review, we challenge the hypothesis that the different treatment alternatives have identical results when applied to different grades of SUI severity, not only in the complete correction of SUI (i.e., the dry rates) but also in the improvement rates. To this end, we investigate the impact of different surgical interventions on outcomes stratified by severity type, using all available current interventions and including every standardized (older and contemporary) instruments of evaluation of SUI severity. This may be already historically proven in the case of severe incontinence in the form of the group of patients with low VLPP/maximal urethral closure pressure; however, current practice utilizes different evaluation tools and different treatment approaches, and contemporary research has not confirmed that the more severe the SUI, the more aggressive treatment is necessary because of increased treatment inefficacy.

### Objectives

The aim of this review is to conduct a systematic evaluation of the success rates and/or dry rates following interventional treatment in patients with mild, moderate, and severe SUI, and compare the results among the various treatments.

## Materials and Methods

### Protocol and Registration

This systematic review was conducted in accordance with the Preferred Reporting Items for Systematic Reviews and Meta-Analyses (PRISMA; Supplementary Table 1) [10]. The study protocol has been registered in the Prospective Register of Systematic Reviews System (PROSPERO ID: CRD420251017900).

### Eligibility Criteria

The Population, Intervention, Comparison, Outcomes, and Study design (PICOS) criteria were utilized for the selection of studies [10].

#### Population

Adult women who underwent an interventional procedure for SUI and had pre- and post-interventional classification of the severity of their SUI. The studies should include women with SUI with standardized evaluation of SUI severity pre- and post-treatment, and describe the results per pre-operative SUI severity group. All patients should be classified clearly according to the severity of SUI, i.e., there was a report of at least a subgroup of patients with mild, moderate, or severe/very severe SUI, as per the instrument of SUI severity classification that was used in each study. A clear definition of the success of the anti-incontinence procedure should be stated in each study. Similarly, the definition of SUI severity should be clearly stated; thus, all the studies should include a detailed description of the methods or the instruments used to assess the success rates and to evaluate the grade of SUI severity (see the Outcomes section).

#### Intervention

The exposure was the use of interventional procedures for SUI treatment. Any type of interventional procedure was included: colposuspensions, MUS (retropubic, trans-obturator, single-incision tension-free vaginal tape), pubo-vaginal slings, energy-based devices (intra-urethral/intravaginal laser, radio-frequencies), and injectables.

#### Comparison

Both single-arm studies and comparative studies were included as long as there was a comparison of success/dry rates, and/or the frequencies according to the pre- and post-interventional grading of SUI after each type of intervention.

#### Outcomes

The data from pre- and post-operative evaluation, such as the number of patients who had different grades of pre- and post-interventional SUI, the cure rates, the dry rates, and the rates of different grades of pre- and post-interventional SUI, had to be clearly presented. The evaluation of grading of SUI could be performed either objectively (urodynamics/VLPP, 1-h/24-h/48-h pad test, International Continence Society (ICS) cough stress test, 1-3-5 cough stress test) or subjectively (IIQ-7, UDI-6, ICIQ, King’s Health Questionnaire [KHQ], Incontinence Quality of Life instrument, Sandvik scale, Ingelman–Sundberg scale, Stamey’s questionnaire, Medical, Epidemiological and Social Aspects of Ageing [MESA] questionnaire). As mentioned above, the population of each study should be clearly classified in subgroups of patients with mild, moderate, or severe/very severe SUI, both pre- and post-operatively, according to the instrument that was used. Any use of nonstandardized evaluation tools of the severity of SUI was considered inadequate design and the study was rejected from further analysis.

#### Study Design

In order to gather any available information, the design included prospective randomized controlled trials (RCTs), prospective cohort studies, and retrospective studies. Thus, this review included studies that compared either single cohorts or comparative head-to-head interventions for SUI with pre- and/or post-operative evaluation of the severity of SUI.

### Information Sources

The present review was conducted by searching (by two independent reviewers: N.R. and I.T.) the three primary online medical databases: MEDLINE database (PubMed), EMBASE database (Scopus), and the Cochrane Library Database (from their official homepage) ever published until 31 December 2024.

Searching through the gray literature, including article abstracts from major international conferences, including the ICS annual congress, the International Urogynecological Association annual meeting, the European Urogynecological Association annual meeting, the American Urogynecology Society annual meeting, and the annual congress of the Federation of International Gynecologists and Obstetricians, was performed online and manually, in order to gather comprehensive results and avoid missing any significant data. Additional studies were identified from searching the reference lists of the studies included.

### Search Strategy

The search methodology employed both free-text keywords and Medical Subject Heading terms, incorporating synonyms and relevant terms. The review team validated the search strategy using the Peer Review of Electronic Search Strategies checklist [11].

### Study Selection Process

Two reviewers (N.R. and I.T.) independently screened the studies retrieved by the literature search. The selection process involved initially screening based on titles and abstracts, followed by a full-text review. Any discrepancy was resolved by discussion with involvement of a third author (T.M.). No health librarian was available to assist in the search.

Studies were excluded if:

1. No report of pre-operative and post-operative severity of SUI was found
2. SUI was recurrent
3. Nonstandardized techniques were used
4. A technique not currently available was used
5. The follow-up period was less than 3 months
6. They had male participants
7. They were not in English

Rayyan online platform was used for the management and screening of the references [12].

### Data Extraction/Data Collection Process/Data Items

Two researchers (N.R. and I.T.) reviewed all the eligible studies and participated in the data collection. Microsoft Excel® was utilized and data forms were created and filled with data obtained from the selected original studies. Separate data tables were generated for each publication with a string of multiple data that assisted in the creation of a consolidated table encompassing all the studies included. The relevant data were extracted by two independent reviewers (N.R. and I.T.) using the data extraction forms. Discrepancies that emerged in relation to collected data were resolved through a process involving consensus among the assessors and consultation with a third investigator (T.M.).

The information extracted from the studies included: study information (authors, year of publication, journal of publication, digital object identifier number, type of study, type of analysis, sample size, location of the study); exposure characteristics (the type of assessment tools used to evaluate SUI severity pre-operatively, the type of intervention, the type of assessment tools used to evaluate SUI severity post-operatively); and outcomes (the number, the type, and the frequency of assessment tools used to evaluate SUI severity). The main outcome of interest was to investigate the success rates and/or dry rates following interventional treatment in patients with mild, moderate, and severe SUI, and compare the results among the various treatments; the secondary outcome was to compare the results of interventional procedures according to the severity of SUI.

In studies where patients with severe pre-operative SUI were included and the post-operative results were reported as ‘dry,’ ‘improved,’ or ‘failure,’ the ‘dry’ group was arbitrarily classified as ‘success’ and the ‘improved’ group was classified as mild/grade I SUI.

### Risk of Bias and Overall Quality of Evidence/Study Risk of Bias Assessment

Quality of assessment for RCTs was evaluated according to the ROB-2 tool, for nonrandomized studies it was evaluated according to the Risk Of Bias in Non-randomized Studies-of interventions (ROBINS-I), and for the rest of the studies it was evaluated according to the Newcastle–Ottawa Scale (NOS). Two independent reviewers (T.M. and N.R.) assessed each paper to verify and confirm the risk of bias.

### Synthesis Methods

The basic information derived from the studies were organized in a Synthesis table. The results from pre-operative evaluation of severity of SUI and post-operative outcomes according to the severity of SUI were organized in two different tables: in the first table the precise number of cured and incontinent patients were allocated according to the pre-operative severity of SUI; in the second table the cured/improved patients were allocated as percentages.

### Statistical Analysis

Basic statistics (means [standard deviations] and medians [ranges]) were calculated using Microsoft Excel®. Any comparisons between mean values (z-scores) were performed using JASP software [13]. No further meta-analysis was performed as there was significant heterogeneity of data.

## Results

### Description of Preferred Reporting Items for Systematic Reviews and Meta-Analyses Flow Chart/Study Selection

**Fig. 1:**
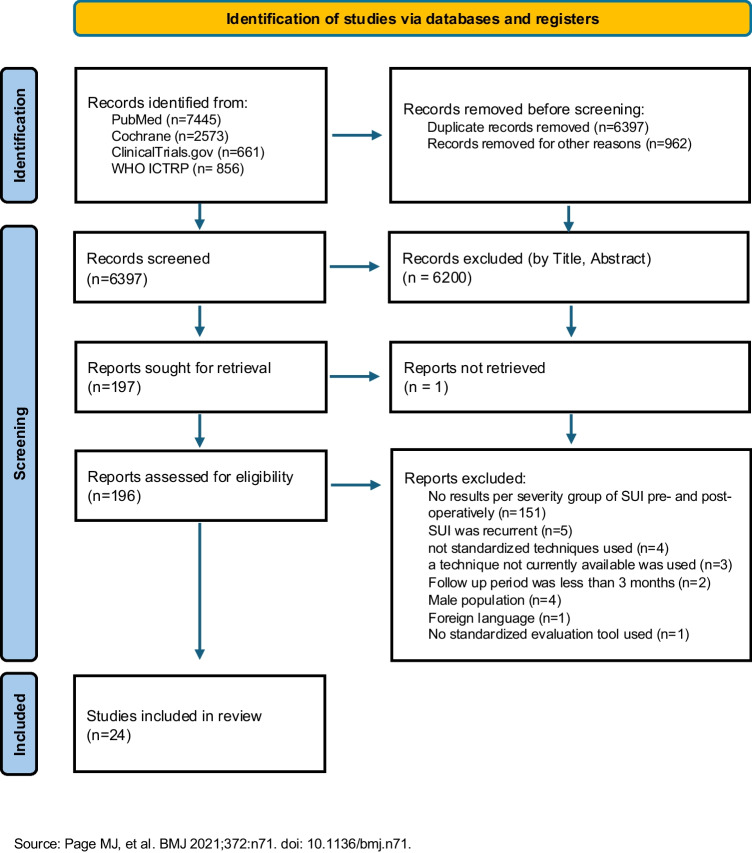
Preferred Reporting Items for Systematic Reviews and Meta-Analyses flow chart

A total of 11,535 articles were found: PubMed: 4039, Cochrane: 3666, ClinicalTrials.gov: 325, WHO ICTRP: 856; Appendix 1). After removing the duplicate entries (6397), we excluded 197 articles based on title and abstract content. Another 172 articles were excluded based on exclusion criteria (PRISMA, Fig. 1, Appendix 1).

### Study Characteristics

Finally, a total of 24 studies (4380 patients) were included for further analysis. Of these, 6 were RCTs (1472 patients) [14–19], 11 were prospective nonrandomized studies (*n* = 543) [20–30], and 7 were retrospective cohort studies (*n* = 1937) [31–37]. In 13 studies (*n* = 3599) a synthetic or autologous graft was used (TVT 6 studies, trans-obturator vaginal tape/outside–in 2 studies, trans-obturator vaginal tape/inside–out (5 studies), single-incision mini-slings (SIMS; 2 studies), autologous/fascia lata sling (1 study)]; in 11 studies (*n* = 781) energy-based devices (erbium-doped yttrium aluminium garnet [Er:YAG] laser 5 studies) CO_2_ laser 3 studies, radio-frequencies (2 studies), or injectables (polydimethylsiloxane 1 study, platelet-rich plasma 1 study) were used.

### Critical Appraisal Within Sources of Evidence/Risk of Bias in Studies

**Fig. 2:**
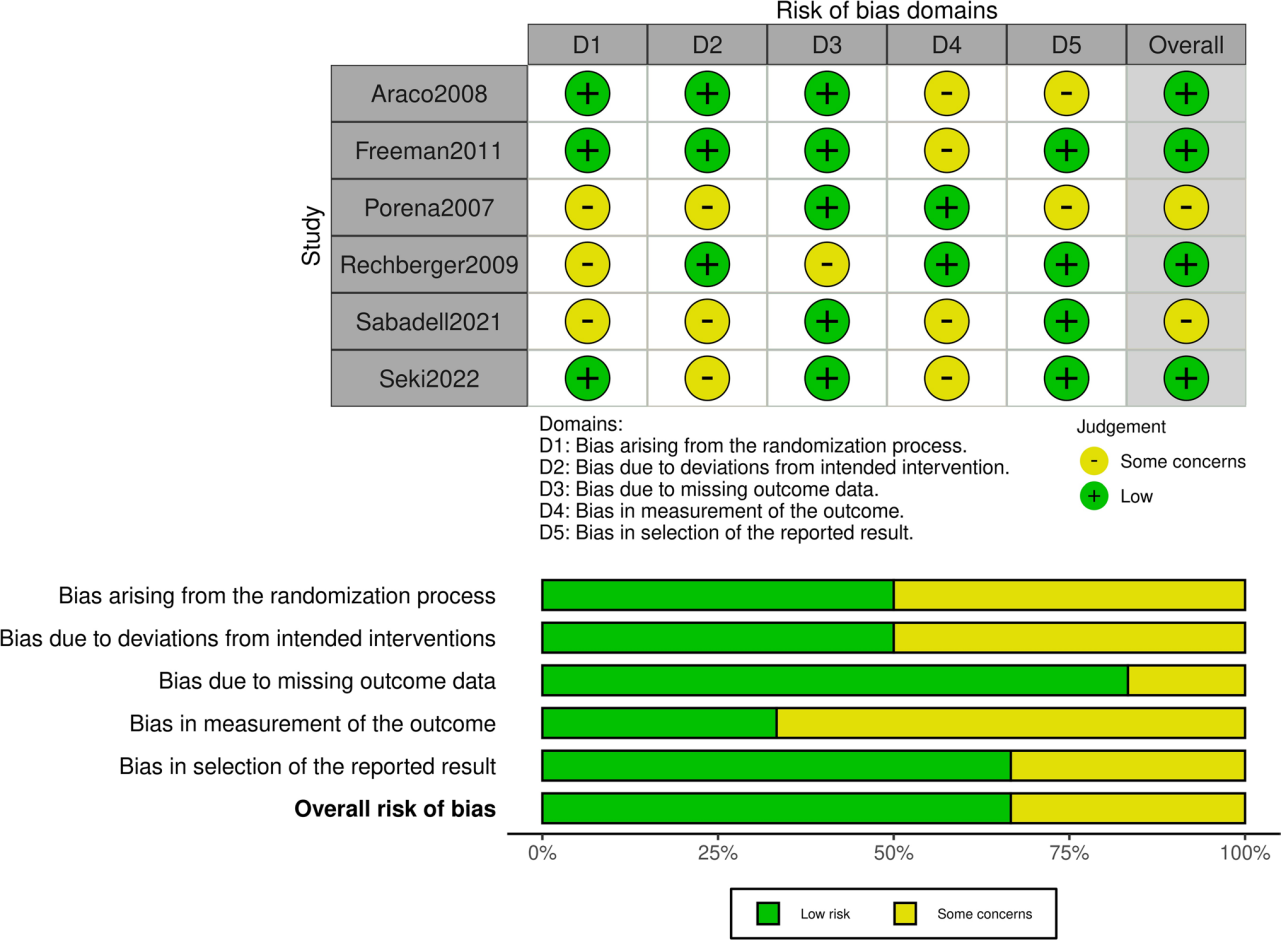
**a** Risk of bias summary for randomized controlled trials (RCTs)/evaluation with the ROB-2 tool. **b** Risk of bias graph for RCT/evaluation with the ROB-2 tool

**Fig. 3:**
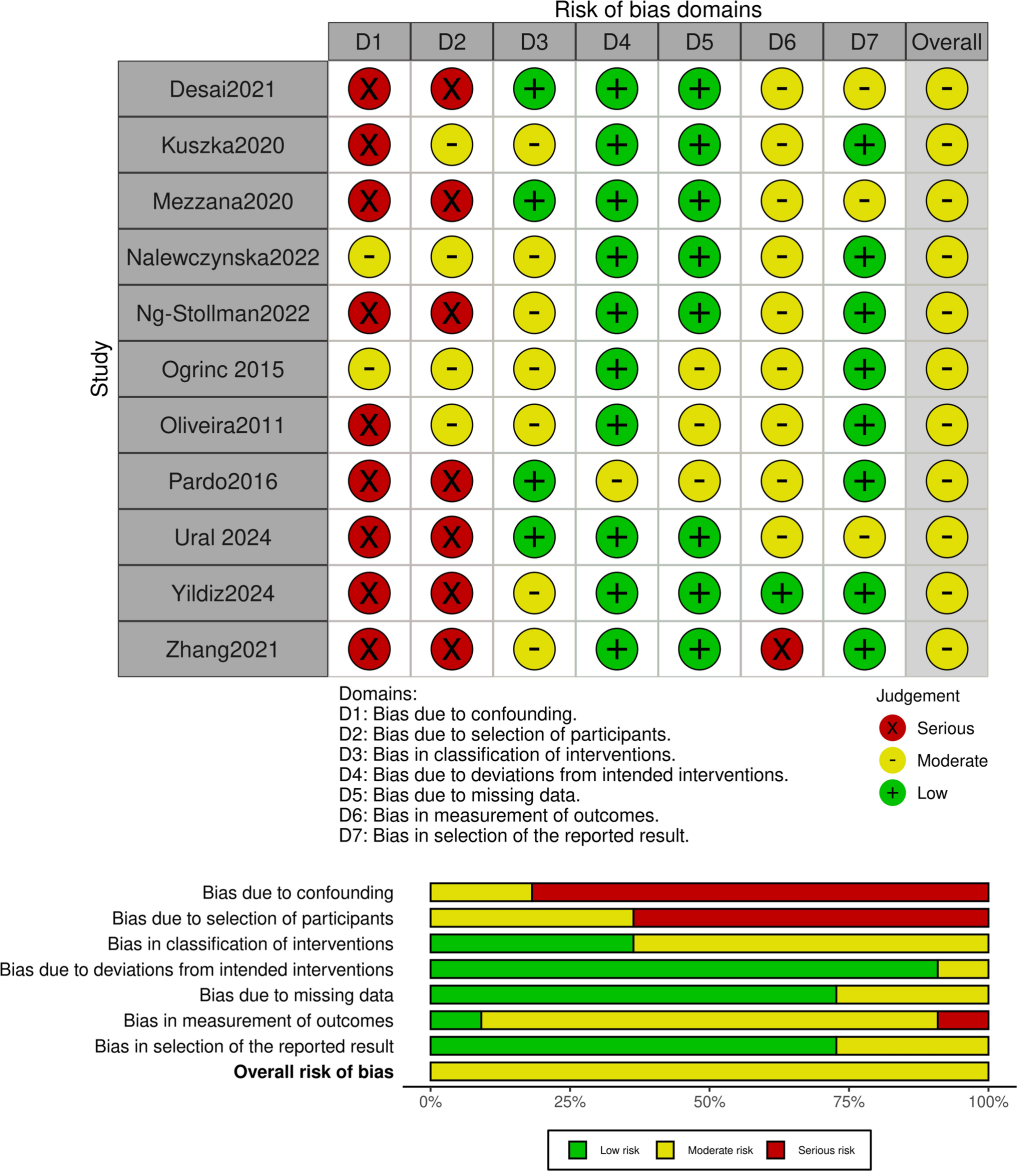
**a** Risk of bias summary for randomized controlled trials (RCTs)/evaluation with the ROBINS-I tool. **b** Risk of bias graph for RCT/evaluation with the ROBINS-I tool

The “Risk of bias summary” (Figs. 2a, 3a) summarizes the reviewers'judgements about risk of bias item for each included study. The “Risk of bias graphs” (Figs. 2b, 3b) illustrate the risk of bias of each category as percentages.

As stated above, RCTs (*n* = 6) were evaluated using the ROB-2 tool, non-RCTs and prospective studies (*n* = 11) were evaluated using the ROBINS-I tool, and retrospective studies (*n* = 13) were evaluated using the NOS.

Regarding the RCTs, 40 (54.1%) of the studies appear to have a low risk of bias, whereas the remaining 34 (45.9%) appear to have some concerns regarding bias (Fig. 2). Of the non-RCTs, 7 (26.9%) appear to have a serious risk of bias, 16 (69.2%) appear to have some concerns regarding bias, and 1 (3.8%) appears to have a serious risk of bias (Fig. 3).

### Results of Individual Sources of Evidence/Results of Individual Studies

The studies included are depicted in Table 1, in which the study design, the study time, the type of intervention, the number, and the mean age of the patients, as well as the type of instruments used for evaluation of the severity of SUI in each study are described (Table 1). Geographically, the studies were conducted in Europe (16 studies), Asia (4 studies), North America (3 studies), and South America (2 studies).

**Table 1:** Synthesis of results

Reference	Study design	Location	Study time	Follow-up (months)	Intervention 1	*n*	Age	Intervention 2	*n*	Age	Severity definition tool	
											Objective	Subjective
Slings												
Freeman et al. [15]	RCT	UK		12	TVT	85	50	TOT	95	54	–	ICIQ-FLUTS Q8a
Frey et al. [34]	Retrospective	Switzerland	2017–2020	2	TVT	168	55.4	–	–	–	–	Ingelman–Sundberg
Porena et al. [16]	RCT	Italy	2002–2005	35	TVT	73	61.8	TOT	75	60.6	–	Ingelman–Sundberg
Rechberger et al. [17]	RCT	Poland	2003–2005	18	Retropubic	269	55.6	TOT	268	55.7	–	Stamey grading
Glass et al. [36]	Retrospective	USA	2005–2012	22	MUS	584	51.7	–	–	–	–	SEAPI
Ng-Stollmann et al. [24]	Prospective	Germany	2012–2019	12	MUS	662	N/A	–	–	–	–	ICIQ-UI-SF
Araco et al. [14]	Prospective	Italy	2004–2006	12	TVT-O	100	53.2	TVT	108	53.6	McGuire Classification	–
Chun et al. [32]	Retrospective	Korea	2004–2006	75	TOT	129	53.6	TVT-O	86	54.4	–	Stamey grading
Sabadell et al. [18]	RCT	Spain	2016–2018	12	TOT/PP	140	55.3	TOT/PVDF	145	58.9	–	Sandvik
Oliveira et al. [26]	Prospective random	Portugal	N/A	12	Mini-Arc	105	52.0	–	–	–	Number of pads	–
Yildiz et al. [29]	Prospective	Turkey	2011–2012	36	SIMS	173	51.0	–	–	–	–	Ingelman–Sundberg
Athanasopoulos et al. [31]	Retrospective	UK	2002–2005	36	Autologous PVS	264	53.0	–	–	–	Number of pads	–
Energy-based devices												
Nalewczynska et al., [23]	Prospective	Poland	2018–2019	12	CO_2_ Laser	60	51.0	–	–	–	–	Sandvik
Zhang et al. [30]	Prospective	China	2018	33	CO_2_ Laser	33	43.1	–	–	–	–	Ingelman–Sundberg
Erel et al. [33]	Retrospective	Turkey	2017–2022	12	Er:YAG laser (IU/IV)	60	56.1	Er:YAG (IV)	62	54.8	–	ICIQ-UI-SF
Kuszka et al. [21]	Prospective	Germany	N/A	28	Er:YAG laser	59	48	–	–	–	1-h pad test	–
Ogrinc et al. [25]	Prospective	Slovenia	2012–2013	12	Er:YAG laser	175	49.7	–	–	–	–	ICIQ-UI-SF
Pardo et al. [27]	Prospective	Chile	2014–2015	5	Er:YAG laser	42	46.5	–	–	–	–	ICIQ-UI-SF
Mezzana [22]	Prospective	Italy	2017	12	Laser	40	56.4	–	–	–	–	Modified SUI scale
Seki et al. [19]	Prospective	Brazil	2018–2019	12	Laser	38	50.2	RF	38	49.2	1-h pad test	–
Mezanna et al. [37]	Retrospective	Italy	2019–2020	3	RF	54	50.7	–	–	–	–	ICIQ-UI-SF
Desai et al. [20]	Prospective	USA	2017–2020	5	RF	48	48.9	–	–	–	–	ICIQ-UI-SF
Injectables												
Ghoniem et al. [35]	Retrospective	USA	2008–2015	36	Macroplastique®	70	63.3	–	–	–	–	Stamey grading
Ural [28]	Prospective	Turkey	2021–2022	6	iPRF	34	51.5	–	–	–	–	ICIQ-UI-SF

*Er:YAG (IU/IV)* erbium-doped yttrium aluminium garnet intra-urethral/intravaginal, *ICIQ-FLUTS* International Consultation Incontinence Questionnaire, Female Lower Urinary Tract Symptoms, *ICIQ-UI-SF* International Consultation Incontinence Questionnaire Urinary Incontinence Short Form, *iPRF* injectable platelet-rich fibrin, *MUS* mid-urethral sling, *N/A* non-applicable, *PP* polypropylene, *PVDF* polyvinylidene difluoride, *PVS* pubo-vaginal sling, *RCT* randomized Controlled Trial, *RF* radio-frequencies, *SEAPI* stress-related leakage, emptying ability, anatomy, protection, inhibition,, *SIMS* single-incision mini-slings, *SUI* stress urinary incontinence, *TOT* trans-obturator tension-free vaginal tape (out–in), *TVT* retropubic tension-free vaginal tape, *TVT-O* trans-obturator tension-free vaginal tape (in–out)

Regarding the instruments that were used for the evaluation of the severity of urinary incontinence peri-operatively, the objective evaluation was performed with the use of the 1-h pad test (2 studies) [19, 21], with the number of pads (2 studies) [26, 31], and with the McGuire classification (1 study) [14]. The subjective evaluation of the peri-operative severity of SUI was performed with the use of International Consultation Incontinence Questionnaire Urinary Incontinence Short Form (ICIQ-UI-SF; 7 studies) [20, 24, 25, 27, 28, 37, 38], with the Ingelman–Sundberg scale or its modifications (4 studies) [16, 29, 30, 34], with the Stamey grading scale (3 studies) [17, 32, 35], with the Sandvik Severity Index (2 studies) [18, 23], with the stress-related leakage, emptying ability, anatomy, protection, inhibition (SEAPI; 1 study) [36], and with a modified SUI scale (1 study) [22].

### Results of Syntheses

According to the results of these studies, successful treatment of mild or grade 1 incontinence was achieved in 84.73% of the patients (333 out of 393; 89.64%, 277 out of 309 patients who had MUS, and 66.67%, 56 out of 84 who had energy-based devices [EBDs]), successful treatment of moderate or grade 2 incontinence in 88.35% of the patients (1145 out of 1296; 92.01%, 1082 out of 1176 patients who had MUS, and 52.50%, 63 out of 120 who had an EBD), and successful treatment of severe/very severe or grade 3 incontinence in 75.73% of the patients (447 out of 591; 83.94%, 392 out of 467 patients who had MUS, and 44.35%, 55 out of 124 who had an EBD). However, dry rates (no incontinence) were achieved in 84.73% of the patients with mild or grade 1 SUI (333 out of 393; 89.64%, 277 out of 309 patients who had MUS, and 66.67%, 56 out of 84 patients who had an EBD), in 73.53% of the patients with moderate or grade 2 SUI (953 out of 1296; 78.32%, 921 out of 1176 patients who had MUS, and 26.67%, 32 out of 120 who had an EBD), and in 57.02% of the patients with severe/very severe or grade 3 SUI (337 out of 591; 70.66%, 330 out of 467 patients who had MUS, and 5.65%, 7 out of 124 patients who had an EBD). There was a statistically significant difference between grade 1, grade 2, and grade 3 success rates (grade 1 vs grade 3, *p* = 0.00056; grade 2 vs grade 3, *p* < 0.0001), but not between grade 1 and grade 2 success rates (grade 1 vs grade 2, *p* = 0.05744), and a statistically significant difference between grade 1, grade 2, and grade 3 dry rates (grade 1 vs grade 2, *p* = 0.0001; grade 1 vs grade 3, *p* < 0.00001; and grade 2 vs grade 3, *p* < 0.00001; Table 2).

**Table 2:** Results of interventions for stress urinary incontinence (SUI): the post-operative results are reported based on pre-operative grades of SUI severity

Reference	Severity definition tool	Follow-up (months)	Intervention	*n*	Success (*n*)	Success (%)	No SUI (*n*)	No SUI (%)	SUI grade I (*n*)	SUI grade II (*n*)	SUI grade III (*n*)
Grade 1											
Porena et al. [16]	Ingelman–Sundberg	35	TVT	3	3	100	3	100	–	–	–
	Ingelman–Sundberg	35	ΤΟΤ	8	7	85	7	85	1	–	–
Rechberger et al. [17]	Stamey grading	18	TVT	35	32	91	32	91	3	–	–
	Stamey grading	18	ΤΟΤ	54	46	85	46	85	6	2	–
Araco et al. [14]	McGuire classification	12	TVT	50	50	100	50	100	–	–	–
	McGuire classification	12	TVT-O	50	50	100	50	100	–	–	–
Glass et al. [36]	SEAPI	22	MUS	88	70	80	70	80	17	1	–
Oliveira et al. [26]	Number of pads	12	Mini-Arc	16	15	93	15	93	1	–	–
Athanasopoulos et al. [31]	Number of pads	36	Autologous PVS	5	4	80	4	80	1	–	–
Subtotal MUS				309	277	90	277	90	29	3	0
Desai et al. [20]	ICIQ-UI-SF	5	RF	8	5	62	5	62	3	–	–
Erel et al. [33]	ICIQ-UI-SF	12	Er:YAG laser (IU/IV)	4	3	75	3	75	1	–	–
Kuszka et al. [21]	1-h pad test	28	Er:YAG laser	32	22	78	22	78	3	7	–
Seki et al. [19]	1-h pad test	12	Laser	18	12	66	12	66	6	–	–
	1-h pad test	12	RF	22	14	64	14	64	8	–	–
Subtotal EBDs				84	56	67	56	67	21	7	0
Grade 1 total				393	333	85	333	85	50	10	0
Grade 2											
Frey et al. [34]	Ingelman–Sundberg	2	TVT	124	116	94	116	94	–	8	–
Porena et al. [16]	Ingelman–Sundberg	35	TVT	54	53	96	36	67	17	1	–
	Ingelman–Sundberg		TOT	54	51	91	40	74	11	3	–
Rechberger et al. [17]	Stamey grading	18	TVT	99	89	90	72	72	17	10	–
	Stamey grading		ΤΟΤ	102	91	89	74	73	17	11	–
Glass et al. [36]	SEAPI	22	MUS	364	341	94	267	73	74	23	–
Araco et al. [14]	McGuire classification	12	TVT-O	50	34	68	34	68	–	17	–
	McGuire classification		TVT	58	58	100	58	100	–	–	–
Oliveira et al. [26]	Number of pads	12	Mini-Arc	79	74	94	74	94	–	5	–
Chun et al. [32]	Stamey grading	75	TOT	74	72	98	63	85	9	2	–
	Stamey grading		TVT-O	54	45	84	36	67	9	–	9
Athanasopoulos et al. [31]	Number of pads	36	Autologous PVS	64	58	80	51	80	7	6	–
Subtotal MUS				1176	1082	92	921	78	161	86	9
Erel et al. [33]	ICIQ-UI-SF	12	Er:YAG laser (IU/IV)	16	10	62	4	25	6	6	–
	ICIQ-UI-SF		Er:YAG laser (IV)	30	16	53	6	20	10	14	–
Kuszka et al. [21]	1-h pad test	28	Er:YAG laser	16	8	50	4	25	4	8	–
Seki et al. [19]	1-h pad test	12	Laser	20	7	35	7	35	13	–	–
	1-h pad test		RF	16	7	44	7	44	9	–	–
Desai et al. [20]	ICIQ-UI-SF	5	RF	15	10	67	4	27	6	5	–
Ural [28]	ICIQ-UI-SF	6	iPRF	7	5	71	–	0	5	2	–
Subtotal EBDs				120	63	52	32	27	53	35	–
Grade 2 total				1296	1145	88	953	73	214	121	9
Grade 3											
Frey et al. [34]	Ingelman–Sundberg	2	TVT	36	31	86	31	86			5
Porena et al. [16]	Ingelman–Sundberg	35	TVT	16	14	54	9	56	5	1	1
	Ingelman–Sundberg		TOT	11	9	70	8	72	1	2	
Rechberger et al. [17]	Stamey grading	18	TVT	48	43	90	32	67	11		5
	Stamey grading		ΤΟΤ	41	31	76	26	63	5		10
Glass et al. [36]	SEAPI	22	MUS	90	77	86	56	62	21	10	3
Chun et al. [32]	Stamey grading	75	TOT	15	14	98	13	87	1		1
	Stamey grading		TVT-O	5	4	84	3	60	1		1
Oliveira et al. [26]	Number of pads	12	Mini-Arc	10	7	70	7	70		0	3
Athanasopoulos et al. [31]	Number of pads	36	Autologous PVS	195	162	74	145	74	17		33
Subtotal MUS				467	392	84	330	71	62	13	62
Erel et al. [15]	ICIQ-UI-SF	12	Er:YAG laser (IU/IV)	44	18	41	2	5	16	22	4
	ICIQ-UI-SF		Er:YAG laser (IV)	28	8	28	3	11	5	14	6
Kuszka et al. [21]	1-h pad test	28	Er:YAG laser	11	0	0	0	0		0	11
Desai et al. [20]	ICIQ-UI-SF	5	RF	14	12	86	2	14	2	8	2
Ural [28]	ICIQ-UI-SF	6	iPRF	27	17	63	0	0	0	17	10
Subtotal EBDs				124	55	44	7	6	23	61	33
Grade 3 total				591	447	76	337	57	85	74	95

*EBDs* energy-based devices, *Er:YAG (IU/IV)* erbium-doped yttrium aluminium garnet intra-urethral/intravaginal, *ICIQ-UI-SF* International Consultation Incontinence Questionnaire Urinary Incontinence Short Form, *iPRF* injectable platelet-rich fibrin, *MUS* mid-urethral sling, *PVS* pubo-vaginal sling, *RF* radio-frequencies, *SEAPI* stress-related leakage, emptying ability, anatomy, protection, inhibition, *TOT* trans-obturator tension-free vaginal tape (out–in), *TVT* retropubic tension-free vaginal tape, *TVT-O* trans-obturator tension-free vaginal tape (in–out)

A similar trend was found with both the objective and the subjective evaluation of SUI severity: the approach with the objective tools showed 86.53%, 81.19%, and 78.24% success rates in grade 1, grade 2, and grade 3 SUI respectively; the approach with the subjective tools showed 83.00%, 89.93%, and 71.20% success rates in grade 1, grade 2, and grade 3 SUI respectively.

According to our results, there was a statistically significant difference regarding the success rates between treatment of grade 1 SUI with MUS in comparison with treatment with energy-based devices (277 out of 309 vs 56 out of 84, *p* < 0.00001). Similarly, there was a statistically significant difference between treatment of grade 2 and grade 3 SUI with MUS in comparison with treatment with energy-based devices (1082 out of 1176 vs 57 out of 120, *p* < 0.00001; 381 out of 467 vs 55 out of 124, *p* < 0.00001).

The improvement in severity scores after treatment is depicted in Table 3. Overall, pre-operatively, 1.17% of patients had no SUI, 9.39% had mild (or grade I) SUI, 35.59% had moderate (or grade II) SUI, 49.39% had severe (or grade III) SUI, and 11.56% had very severe SUI. Post-intervention, 46.20% of the patients were dry, 24.86% had mild (or grade I) SUI, 27.04% had moderate (or grade II) SUI, 7.93% had severe (or grade III) SUI, and 1.34% had very severe SUI (Table 3).

**Table 3:** Results of interventions for stress urinary incontinence (SUI): the post-operative results are reported based on pre-operative grades of SUI severity

Reference	Severity definition tool	Follow-up (months)	Intervention 1	*n*	No SUI		Mild SUI/grade I		Moderate SUI/grade II		Severe SUI/grade III		Very severe SUI	
					Pre-operatively (%)	Post-operatively (%)	Pre-operatively (%)	Post-operatively (%)	Pre-operatively (%)	Post-operatively (%)	Pre-operatively (%)	Post-operatively (%)	Pre-operatively (%)	Post-operatively (%)
Erel et al. [33]	ICIQ-SF	12	Er:YAG laser (IU/IV)	60	0.0	10.0	0.0	36.7	26.7	46.7	58.3	5.0	15.0	1.7
	ICIQ-SF	12	Er:YAG laser (IV)	62	0.0	19.4	6.5	25.8	48.4	45.2	38.7	8.1	6.5	1.6
Freeman et al. [15]	ICIQ-FLUTS Q8a	12	TVT	92	2.2	65.5	8.7	26.1	28.3	5.4	60.8	4.3	N/A	N/A
	ICIQ-FLUTS Q8a	12	TOT	98	1.0	63.4	5.0	34.7	31.0	2.1	61.0	8.1	N/A	N/A
Ghoniem et al. [35]	Stamey grading	36	Macroplastique®	70	0.0	30.0	37.1	40.0	54.3	27.1	8.6	2.8	0.0	0.0
Mezzana. [22]	Modified SUI scale	12	Laser system	40	25.0	70.0	45.0	25.0	25.0	0.0	5.0	5.0	0.0	0.0
Mezzana et al. [37]	ICIQ-SF	3	RF	54	0.0	0.0	13.0	65.0	61.0	33.0	26.0	2.00	0.0	0.0
Nalewczynska et al. [23]	Sandvik	12	Pixel CO_2_ laser	59	0.0	0.0	2.0	25.0	73.0	75.0	25.0	0.0	0.0	0.0
Ng-Stollmann et al. [24]	ICIQ-SF	12	MUS	662	0.0	32.2	0.8	20.2	26.1	31.3	58.8	13.3	14.4	3.0
Ogrinc et al. [25]	ICIQ-SF	12	Er: YAG laser	175	0.0	62.0	17.0	24.0	27.0	12.0	51.0	2.0	5.0	0.0
Pardo et al. [27]	ICIQ-SF	5	Er: YAG laser	42	0.0	38.1	9.5	26.2	47.6	23.8	40.5	11.9	2.4	0.0
Sabadell et al. [18]	Sandvik	12	TOT PP	134	2.9	54.5	2.2	15.2	19.0	24.2	46.7	5.30	29.2	0.8
	Sandvik	12	TOT PVDF	137	2.8	55.9	0.7	17.6	16.3	23.5	46.8	2.2	33.3	0.8
Ural [28]	ICIQ-SF	6	iPRF	34	0.0	0.0	0.0	14.7	20.6	55.9	73.5	29.4	5.9	0.0
Yildiz et al. [29]	Ingelman–Sundberg	36	Single-incision sling	173	0.0	83.8	30.6	6.4	57.2	9.8	12.2	0.0	0.0	0.0
Zhang et al. [30]	Ingelman–Sundberg (modified)	33	FemiLift CO_2_ laser	18	0.0	48.4	16.7	45.4	83.3	6.1	0.0	0.0	0.0	0.0
Total					1.1	43.3	8.8	23.3	33.3	25.3	46.3	7.4	10.8	1.3

*Er:YAG (IU/IV)* erbium erbium-doped yttrium aluminium garnet intra-urethral/intravaginal, *ICIQ-FLUTS* International Consultation Incontinence Questionnaire, Female Lower Urinary Tract Symptoms, *ICIQ-UI-SF* International Consultation Incontinence Questionnaire Urinary Incontinence Short Form, *iPRF* injectable platelet-rich fibrin, *MUS* mid-urethral sling, *PP* polypropylene, *PVDF* polyvinylidene difluoride, *RF* radio-frequencies, *SEAPI* stress-related leakage, emptying ability, anatomy, protection, inhibition, *SUI* stress urinary incontinence, *TOT* trans-obturator tension-free vaginal tape (out–in), *TVT* retropubic tension-free vaginal tape, *TVT-O* trans-obturator tension-free vaginal tape (in–out)

Further meta-analysis could not be performed due to the significant heterogeneity of the methodology and the reporting of the outcomes of the studies.

## Discussion

This study demonstrates that, according to the best available evidence and in patients with uncomplicated primary SUI, current treatments of mild, moderate, and severe SUI, have an average success rate of 83.64%, 78.20%, and 70.15% respectively. Consequently, this study concludes that the outcome of SUI interventional treatment depends largely on the grade of SUI severity. The data derive from studies of variable and mainly low levels of evidence (from prospective RCTs to retrospective cohort studies), although the major bias is the different instrument tools that have been used to evaluate the severity of incontinence among the various studies.

### What This Study Adds?

To our knowledge, this is the first review that offers information about the success of SUI treatment according to the grade of pre-operative incontinence severity. Our study accumulates evidence in order to assist in the process of counseling those patients who wish to undergo intervention for SUI, comparing data from different treatment modalities based on the efficacy of each method in the correction of incontinence according to the pre-operative severity grading. The study suggests that there is significant difference between treatment modalities and that the more severe the incontinence, the more aggressive the intervention should be in order to accomplish dryness. The study suggests that MUS perform consistently better than nonsurgical alternatives (i.e., energy-based devices or platelet-rich fibrin) in the treatment of SUI. However, the more severe the incontinence, the less efficacious the methods (both MUS and EBDs) are. Moreover, the same studies indicate that when improvement is the main outcome, all methods have acceptable results in the groups of patients with mild and moderate SUI.

In detail, mild SUI is treated in 89.64% of the patients with an MUS compared with 66.67% of those with an EBD. The latter may be acceptable for a group of patients who wish to avoid the potential complications of the MUS. Moderate SUI is treated in 92.01% of the patients (78.32% achieve dry status) with an MUS, compared with 52.50% with an EBD (26.67% achieve dry status). The application of EBDs in this group may be suboptimal for the majority of patients. The same may apply to the group of patients with severe SUI, 83.94% of whom are treated with an MUS (70.66% achieve dry status) compared with 44.35% with an EBD (5.65% achieve dryness).

It appears that, according to our results, mild SUI can be treated efficiently with both MUS and EBDs, in comparison with moderate or severe SUI, which are optimally treated with an MUS. This conclusion necessitates the pre-operative evaluation of SUI severity with the aid of a reliable, reproducible tool. Ideally, this could be performed both in an objective and in a subjective way, as these different approaches may elicit different results in different subcategories of SUI (i.e., low VLPP, mixed urinary incontinence, older or obese patients, etc.). Our study results highlight the absence of homogeneity in the use of validated instruments for the evaluation of SUI severity in both the academic and the clinical setting. Thus, there is an urgent need for consensus toward universally accepted, easy to use, tools that could evaluate the severity of SUI reliably both pre- and post-operatively.

As mentioned above, our results indicate that the higher the SUI severity, the more difficult the interventional control of symptoms. For example, the dry rates after MUS in cases of severe SUI are marginally higher than 2 out of 3 (70.66%). This fact emphasizes the need for introducing better, more successful treatments, not to mention the need for treatment with a favorable complication profile compared with the current modalities. Vice versa, mild SUI treatment can be achieved in acceptable rates (66.25% dryness) using less invasive techniques in women not willing to accept the complication rates of surgical alternatives.

### Comparison with Previous Studies

Recently, Abdel-Fattah et al. led the completion of the SIMS trial, in which 298 patients with SUI underwent a mini-sling procedure and they were compared with 298 women with SUI who underwent a MUS procedure. Pre-operative evaluation included a 24-h pad test and the ICIQ-UI-SF. Both groups had mainly pre-operative moderate SUI: 39 g (24–60 g) and 40 g (24–67 g) in the 24-h pad test and 14.4 ± 3.3 and 14.4 ± 3.3 ICIQ-UI-SF scores. The study concluded a non-inferiority of SIMS in the treatment of SUI compared with MUS. However, the study may have included a small percentage of patients with severe SUI (24-h pad test > 75 g), where both treatments might have behaved differently [39] and any extrapolation of these results in patients with severe SUI would be inappropriate.

Interestingly, in the Trial Of Mid-Urethral Slings Richter et al. included 597 patients with a median 24-h pad test of 11.5 g in the TVT group and 13.7 g in the TOT group. Both groups consisted mainly of patients with mild urinary incontinence (the classification of 24-h pad test results are: 1.3–4.4 g/no incontinence, 4–20 g/mild incontinence, 21–74 g/moderate incontinence, and 75 g/severe incontinence). The authors concluded that both techniques fulfilled the study’s criteria for success. However, any extrapolation of these results in patients with moderate or severe SUI would be inappropriate [40].

Other significant studies in the field have mainly recruited patients with moderate SUI. For example, Ward et al. studied 344 patients with a 1-h pad test of 18 g (the TVT group) and 16 g (the Burch group) [41]; Rudnicki et al. studied 305 patients with an ICIQ-UI-SF score of 15 ± 5.0 (the SIMS group) and 15 ± 4.0 (the MUS group) [42]; Krofta et al. studied 300 patients with an ICIQ-UI-SF score of 13.3 ± 15.8 (the TVT group) and 13.8 ± 4.8 (the TVT-O group) [43]; Aigmüller et al. studied 584 patients with a KHQ SUI score of 48.7 ± 11.2 (the TVT group) and 48.7 ± 11.5 (the TOT group) [44]. All these studies included a significant portion of patients with mild SUI and severe SUI, but they did not include a stratification of their results according to the pre-operative SUI severity sub-groups; therefore, any significant information about the efficacy of the techniques for patients with mild or severe incontinence remains unavailable.

### How Do the Tools of Evaluation of Stress Urinary Incontinence Severity Compare with Each Other?

In 2007, Albo et al. concluded that urinary incontinence severity measures correlate moderately with each other at best. Clinical tools for the objective evaluation of SUI such as supine empty bladder test and ALPP did not demonstrate a clinically significant association with other severity measures, whereas tools for subjective evaluation of SUI such as the MESA questionnaire) demonstrated stronger correlation with the other measures of subjective evaluation of severity of SUI and quality of life [38]. Since that publication, little knowledge has been added in this field; thus, the observation that although numerous methods have been proposed to measure the severity of SUI, none has emerged as the gold standard still holds true. Apparently, the lack of a universally accepted severity measure creates difficulty in designing or comparing results, both in clinical settings and in research [38].

### Strengths and Limitations

First, a strength of this review is that a very large number of patients (*n* = 4380) are collected from miscellaneous interventional approaches. Second, in the study we attempt to describe and compare results according to realistic outcomes after incontinence surgery. Most studies that report incontinence surgery results follow the binary model of post-operative dry/incontinent patients. Dry rates or success rates are very useful for direct comparisons of the efficacy of a procedure; however, in real life, this approach is replaced by a continuous model of improvement, similar to the PGI-I scale (not improved to very much improved) [45]. Indeed, as there are many women who could choose a less aggressive procedure achieving slightly reduced success rates over an aggressive procedure with better dry rates but increased complication rates, this issue remains constantly interesting, and this study presents a more pragmatic variation of the results of each anti-incontinence procedure.

The main limitation of this review is the low quality of the bulk of the studies included. It is true that the lower the quality of the primary data the poorer the value of the conclusions of any review; however, as this is the first systematic approach to this subject, it was considered necessary to include lower-quality studies such as retrospective cohorts. Another serious limitation of this review is the effect of nonhomogeneity among both the evaluation tools and the treatment modalities. In fact, the ideal review would be to analyze each intervention using a different evaluation tool, but this is of no value, because it leads to recovery of a very small number of studies as the relevant literature is very scanty.

This study is aimed at systematically comparing studies that apply different interventions for the control of SUI. This may be a methodological error, but in real life it is something that happens daily. In fact, the comparison between the success rates after MUS versus EBD in the mild SUI group indicates a possible role for this type of intervention in this group of patients. Thus, our results challenge the current diagnostic and post-operative practice, indicating that the identification of a parameter long undermined, i.e., the severity of SUI, may be crucially important, as its burden defines the post-operative results significantly.

The secondary aim of this review, which was to compare in the context of a meta-analysis the effectiveness of different surgical methods based on the severity of SUI, could not be accomplished owing to insufficient data. Conducting a meta-analysis to compare post-treatment outcomes across varying pre-operative SUI severity levels was unfeasible because of inconsistencies in the assessment tools used to evaluate SUI severity across studies, the absence of pre-operative patient sub-group classifications based on SUI severity, and the lack of reported success rates corresponding to pre-operative SUI severity classification.

Finally, our results cannot address the correlations of the severity of SUI with widely accepted conditions such as intrinsic sphincter deficiency, or other potential risk factors for less favorable surgical outcomes [35]. In the majority of the studies the raw data did not permit an in-depth analysis in order to investigate any such associations [1].

## Conclusion

Overall, this review suggests that the success rates of any incontinence procedure could depend on the pre-operative severity of SUI, and that these success rates are significantly lower with increasing SUI severity. MUS appear to produce better results than EBDs for any SUI severity. EBDs appear to have acceptable success rates for mild SUI. Further studies focusing on the treatment of SUI severity groups are needed to confirm these results.

## Supplementary Information

Below is the link to the electronic supplementary material.

Supplementary file1 (DOCX 17 KB)

## Data Availability

Available from the authors at any request.

## Declarations

### Registration and Protocol

PROSPERO ID: CRD420251017900.

## References

[1] Mikos T, Theodoulidis I, Karalis T, Zafrakas M, Grimbizis GF. Instruments used for the assessment of SUI severity in urogynecologic surgical trials: a scoping review. Int Urogynecol J. 2024;35:2255–79. 10.1007/s00192-024-05934-w. 39425774 10.1007/s00192-024-05934-w

[2] Blaivas JG, Olsson CA. Stress incontinence: classification and surgical approach. J Urol. 1988;139:727–31. 10.1016/s0022-5347(17)42611-5. 3352031 10.1016/s0022-5347(17)42611-5

[3] McGuire EJ, Fitzpatrick CC, Wan J, Bloom D, Sanvordenker J, Ritchey M, et al. Clinical assessment of urethral sphincter function. J Urol. 1993;150:1452–4. 10.1016/s0022-5347(17)35806-8. 8411422 10.1016/s0022-5347(17)35806-8

[4] Ford AA, Ogah JA. Retropubic or transobturator mid-urethral slings for intrinsic sphincter deficiency-related stress urinary incontinence in women: a systematic review and meta-analysis. Int Urogynecol J. 2016;27:19–28. 10.1007/s00192-015-2797-3. 26220506 10.1007/s00192-015-2797-3

[5] NICE guideline. Urinary incontinence and pelvic organ prolapse in women: management. Published 2 April 2019. www.nice.org.uk/guidance/ng123. 31211537

[6] Nager CW, Brubaker L, Litman HJ, Zyczynski HM, Varner RE, Amundsen C, et al. A randomized trial of urodynamic testing before stress-incontinence surgery. N Engl J Med. 2012;366:1987–97. 10.1056/NEJMoa1113595. 22551104 10.1056/NEJMoa1113595 PMC3386296

[7] Sandvik H, Hunskaar S, Seim A, Hermstad R, Vanvik A, Bratt H. Validation of a severity index in female urinary incontinence and its implementation in an epidemiological survey. J Epidemiol Community Health. 1993;47:497–9. 10.1136/jech.47.6.497. 8120507 10.1136/jech.47.6.497 PMC1059866

[8] Schüssler B, Alloussi S. Zur Klassifikation der Stressinkontinenz nach Ingelman-Sundberg. Gynakol Rundsch. 1983;23:166–74. 6642284

[9] Stamey TA. Endoscopic suspension of the vesical neck for urinary incontinence in females. Report on 203 consecutive patients. Ann Surg. 1980;192:465–71. 10.1097/00000658-198010000-00005. 7425693 10.1097/00000658-198010000-00005 PMC1346989

[10] Page MJ, McKenzie JE, Bossuyt PM, Boutron I, Hoffmann TC, Mulrow CD, et al. The PRISMA 2020 statement: an updated guideline for reporting systematic reviews. BMJ. 2021;372:n71. 10.1136/bmj.n71. 33782057 10.1136/bmj.n71 PMC8005924

[11] McGowan J, Sampson M, Salzwedel DM, Cogo E, Foerster V, Lefebvre C. PRESS peer review of electronic search strategies: 2015 guideline statement. J Clin Epidemiol. 2016;75:40–6. 10.1016/j.jclinepi.2016.01.021. 27005575 10.1016/j.jclinepi.2016.01.021

[12] Ouzzani M, Hammady H, Fedorowicz Z, Elmagarmid A. Rayyan—a web and mobile app for systematic reviews. Syst Rev. 2016;5:210. 10.1186/s13643-016-0384-4. 27919275 10.1186/s13643-016-0384-4 PMC5139140

[13] JASP Team. JASP v. 0.19.1 (JASP, 2024).

[14] Araco F, Gravante G, Sorge R, Overton J, De Vita D, Sesti F, et al. TVT-O vs TVT: a randomized trial in patients with different degrees of urinary stress incontinence. Int Urogynecol J Pelvic Floor Dysfunct. 2008;19(7):917–26. 10.1007/s00192-007-0554-y. 18217177 10.1007/s00192-007-0554-y

[15] Freeman R, Holmes D, Hillard T, Smith P, James M, Sultan A, et al. What patients think: patient-reported outcomes of retropubic versus trans-obturator mid-urethral slings for urodynamic stress incontinence–a multi-centre randomised controlled trial. Int Urogynecol J. 2011;22:279–86. 10.1007/s00192-010-1343-6. 21222114 10.1007/s00192-010-1343-6

[16] Porena M, Costantini E, Frea B, Giannantoni A, Ranzoni S, Mearini L, et al. Tension-free vaginal tape versus transobturator tape as surgery for stress urinary incontinence: results of a multicentre randomised trial. Eur Urol. 2007;52:1481–90. 10.1016/j.eururo.2007.04.059. 17482343 10.1016/j.eururo.2007.04.059

[17] Rechberger T, Futyma K, Jankiewicz K, Adamiak A, Skorupski P. The clinical effectiveness of retropubic (IVS-02) and transobturator (IVS-04) midurethral slings: randomized trial. Eur Urol. 2009;56:24–30. 10.1016/j.eururo.2009.02.038. 19285788 10.1016/j.eururo.2009.02.038

[18] Sabadell J, Pereda-Núñez A, Ojeda-de-Los-Santos F, Urbaneja M, González-García C, Camps-Lloveras N, et al. Polypropylene and polyvinylidene fluoride transobturator slings for the treatment of female stress urinary incontinence: 1-year outcomes from a multicentre randomized trial. Neurourol Urodyn. 2021;40:475–82. 10.1002/nau.24586. 33259073 10.1002/nau.24586 PMC7839450

[19] Seki AS, Bianchi-Ferraro AMHM, Fonseca ESM, Sartori MGF, Girão MJBC, Jarmy-Di Bella ZIK. CO2 laser and radiofrequency compared to a sham control group in treatment of stress urinary incontinence (LARF study arm 3). A randomized controlled trial. Int Urogynecol J. 2022;33:3535–42. 10.1007/s00192-022-05091-y. 35254473 10.1007/s00192-022-05091-y

[20] Desai SA, Vakil Z, Kroumpouzos G. Transcutaneous temperature-controlled radiofrequency treatment: improvement in female genital appearance, sexual dysfunction, and stress urinary incontinence. Aesthet Surg J. 2021;41:1400–8. 10.1093/asj/sjab174. 33843969 10.1093/asj/sjab174

[21] Kuszka A, Gamper M, Walser C, Kociszewski J, Viereck V. Erbium:YAG laser treatment of female stress urinary incontinence: midterm data. Int Urogynecol J. 2020;31:1859–66. 10.1007/s00192-019-04148-9. 31828400 10.1007/s00192-019-04148-9

[22] Mezzana P. “Two wavelengths endovaginal laser system”: Clinical evaluation of a new device for mild SUI and vaginal atrophy treatment. Dermatol Ther. 2020;33:e14445. 10.1111/dth.14445. 33098352 10.1111/dth.14445

[23] Nalewczynska AA, Barwijuk M, Kolczewski P, Dmoch-Gajzlerska E. Pixel-CO_2_ laser for the treatment of stress urinary incontinence. Lasers Med Sci. 2022;37:1061–7. 10.1007/s10103-021-03353-7. 34382127 10.1007/s10103-021-03353-7 PMC8918174

[24] Ng-Stollmann N, Fünfgeld C, Hüsch T. The impact of partially absorbable midurethral slings in stress urinary incontinence surgery: a cohort study. Int J Gynaecol Obstet. 2022;158:730–5. 10.1002/ijgo.14095. 35044679 10.1002/ijgo.14095

[25] Ogrinc UB, Senčar S, Lenasi H. Novel minimally invasive laser treatment of urinary incontinence in women. Lasers Surg Med. 2015;47:689–97. 10.1002/lsm.22416. 26388213 10.1002/lsm.22416 PMC5396289

[26] Oliveira R, Botelho F, Silva P, Resende A, Silva C, Dinis P, et al. Single-incision sling system as primary treatment of female stress urinary incontinence: prospective 12 months data from a single institution. BJU Int. 2011;108:1616–21. 10.1111/j.1464-410X.2011.10158.x. 21457429 10.1111/j.1464-410X.2011.10158.x

[27] Pardo JI, Solà VR, Morales AA. Treatment of female stress urinary incontinence with erbium-YAG laser in non-ablative mode. Eur J Obstet Gynecol Reprod Biol. 2016;204:1–4. 10.1016/j.ejogrb.2016.06.031. 27448169 10.1016/j.ejogrb.2016.06.031

[28] Ural ÜM. The effect of injectable platelet rich fibrin as a nonsurgical treatment of the female stress urinary incontinence. Arch Gynecol Obstet. 2024;309:2229–36. 10.1007/s00404-024-07431-3. 38424182 10.1007/s00404-024-07431-3

[29] Yildiz G, Ceylan Y, Ucer O, Arslan D, Çelik O, Gunlusoy B. Safety and efficacy of single-incision sling for female stress urinary incontinence: 3 years’ results. Int Urogynecol J. 2016;27:1667–71. 10.1007/s00192-016-3001-0. 26992728 10.1007/s00192-016-3001-0

[30] Zhang L, Lai Y, Pan W, Zhou B, Qiang X, Mao Z, et al. Application of ultra pulse CO_2_ lattice laser in the treatment of female urinary incontinence. Transl Androl Urol. 2021;10:2471–7. 10.21037/tau-21-337. 34295733 10.21037/tau-21-337 PMC8261411

[31] Athanasopoulos A, Gyftopoulos K, McGuire EJ. Efficacy and preoperative prognostic factors of autologous fascia rectus sling for treatment of female stress urinary incontinence. Urology. 2011;78:1034–8. 10.1016/j.urology.2011.05.069. 22054371 10.1016/j.urology.2011.05.069

[32] Chun JY, Song M, Yoo DS, Han JY, Hong B, Choo MS. A comparative study of outside-in and inside-out transobturator tape procedures for female stress urinary incontinence: 7-year outcomes. LUTS: Lower Urinary Tract Symptoms. 2014;6:145–50. 10.1111/luts.12052. 26663595 10.1111/luts.12052

[33] Erel CT, Gambacciani M, Ozcivit Erkan IB, Gokmen Inan N, Hamzaoglu Canbolat K, Fidecicchi T. SUI in postmenopausal women: advantages of an intraurethral + intravaginal Er:YAG laser. Climacteric. 2023;26:503–9. 10.1080/13697137.2023.2210282. 37211026 10.1080/13697137.2023.2210282

[34] Frey JN, Zellweger M, Krebs J, Christmann C. Impact of defined risk factors on degree of urinary stress incontinence and sling outcome: a retrospective cohort analysis. J Clin Med. 2023;12:5422. 10.3390/jcm12165422. 37629465 10.3390/jcm12165422 PMC10456048

[35] Ghoniem G, Farhan B, Chowdhury ML, Chen Y. Safety and efficacy of polydimethylsiloxane (Macroplastique®) in women with stress urinary incontinence: analysis of data from patients who completed three years follow-up. Int Urogynecol J. 2021;32:2835–40. 10.1007/s00192-021-04827-6. 34100973 10.1007/s00192-021-04827-6 PMC8455384

[36] Glass A, Durbin-Johnson B, Rothschild J, Gomelsky A. Seapi incontinence classification system: 1-year postoperative results following midurethral sling placement. Female Pelvic Med Reconstr Surg. 2020;26:671–6. 10.1097/SPV.0000000000000656. 30418297 10.1097/SPV.0000000000000656

[37] Mezzana P, Garibay I, Fusco I. Vaginal bipolar radiofrequency treatment of mild SUI: a pilot retrospective study. Medicina (Kaunas). 2022;58:181. 10.3390/medicina58020181. 35208505 10.3390/medicina58020181 PMC8878952

[38] Albo M, Wruck L, Baker J, Brubaker L, Chai T, Dandreo KJ, et al. The relationships among measures of incontinence severity in women undergoing surgery for stress urinary incontinence. J Urol. 2007;177:1810–4. 10.1016/j.juro.2007.01.032. 17437826 10.1016/j.juro.2007.01.032

[39] Abdel-Fattah M, Cooper D, Davidson T, Kilonzo M, Hossain M, Boyers D, et al. Single-incision mini-slings for stress urinary incontinence in women. N Engl J Med. 2022;386:1230–43. 10.1056/NEJMoa2111815. 35353961 10.1056/NEJMoa2111815

[40] Richter HE, Albo ME, Zyczynski HM, Kenton K, Norton PA, Sirls LT, et al. Retropubic versus transobturator midurethral slings for stress incontinence. N Engl J Med. 2010(22). 10.1056/NEJMoa0912658. 10.1056/NEJMoa0912658 PMC2962585 20479459

[41] Ward K, Hilton P; United Kingdom and Ireland Tension-free Vaginal Tape Trial Group. Prospective multicentre randomised trial of tension-free vaginal tape and colposuspension as primary treatment for stress incontinence. BMJ. 2002;325:67. 10.1136/bmj.325.7355.67. 12114234 10.1136/bmj.325.7355.67 PMC117136

[42] Rudnicki M, von Bothmer-Ostling K, Holstad A, Magnusson C, Majida M, Merkel C, et al. Adjustable mini-sling compared with conventional mid-urethral slings in women with urinary incontinence. A randomized controlled trial. Acta Obstet Gynecol Scand. 2017;96:1347–56. 10.1111/aogs.13205. 28815547 10.1111/aogs.13205

[43] Krofta L, Feyereisl J, Otcenásek M, Velebil P, Kasíková E, Krcmár M. TVT and TVT-O for surgical treatment of primary stress urinary incontinence: prospective randomized trial. Int Urogynecol J. 2010;21:141–8. 10.1007/s00192-009-1027-2. 19907913 10.1007/s00192-009-1027-2

[44] Aigmüller T, Tammaa A, Tamussino K, Hanzal E, Umek W, Kölle D, et al. Retropubic vs. transobturator tension-free vaginal tape for female stress urinary incontinence: 3-month results of a randomized controlled trial. Int Urogynecol J. 2014;25:1023–30. 10.1007/s00192-014-2384-z. 24819327 10.1007/s00192-014-2384-z

[45] Guy W, editor. ECDEU assessment manual for psychopharmacology. Rockville, MD: US Department of Health, Education, and Welfare Public Health Service Alcohol, Drug Abuse, and Mental Health Administration; 1976.

