# Supplementary material for: Does Pre-Operative Grade of Stress Urinary Incontinence Severity Affect the Post-Operative Outcome? A Systematic Review
Source: Int Urogynecol J. 2025 Sep 24;36(10):1935–49. doi: 10.1007/s00192-025-06275-y (PMC12618355; doi:10.1007/s00192-025-06275-y)
Supplement: Supplementary file 1 — Supplementary file1 (DOCX 17 KB) [file 192_2025_6275_MOESM1_ESM.docx]

Table XXX. NOS score for the cohort studies.

| **Assessment of quality of a cohort study – Newcastle Ottawa Scale** | | | | | | | | | |  |
| --- | --- | --- | --- | --- | --- | --- | --- | --- | --- | --- |
| **Study** | **Selection** | | | | **Comparability** | | **Outcome** | | | **Score** |
|  | Representativeness of the intervention cohort | Selection of the non intervention cohort | Ascertainment of intervention | Demonstration that outcome of interest was not present at start of study | Comparability of cohorts on the basis of the design or analysis |  | Assessment of outcome | Was follow up long enough for outcomes to occur | Adequacy of follow up of cohorts |  |
|  |  |  |  |  | study controls for age, sex, marital status | study controls for any additional factors (e.g. socio-economic status, education) |  |  |  |  |
| Athanasopoulos et al, 2011, Urology | 1 | 1 | 0 | 0 | 0 | 0 | 1 | 0 | 1 | 4 - Fair |
| Chun et al, 2014, Low Urin Tract Symptoms | 1 | 1 | 1 | 1 | 0 | 1 | 1 | 0 | 1 | 7 - Good |
| Erel et al, 2023, Climacteric | 1 | 1 | 1 | 1 | 1 | 1 | 1 | 0 | 1 | 8 - Good |
| Frei et al, 2023, J Clin Med. | 1 | 1 | 0 | 0 | 0 | 1 | 1 | 0 | 1 | 5 - Fair |
| Ghoniem et al, 2021, Int Urogynecol J | 1 | 1 | 0 | 0 | 0 | 1 | 1 | 0 | 1 | 5 - Fair |
| Glass et al, 2020, Female Pelvic Med Reconstr Surg. | 1 | 1 | 0 | 0 | 0 | 0 | 1 | 0 | 0 | 3 - Fair |
| Mezzana et al, 2022, Medicina | 1 | 0 | 1 | 0 | 0 | 0 | 0 | 1 | 1 | 4 - Fair |
